# Application of the Filariasis CELISA Antifilarial IgG_4_ Antibody Assay in Surveillance in Lymphatic Filariasis Elimination Programmes in the South Pacific

**DOI:** 10.1155/2011/492023

**Published:** 2011-09-26

**Authors:** Hayley Joseph, Fuatai Maiava, Take Naseri, Fasihah Taleo, Malakai ‘Ake, Corinne Capuano, Wayne Melrose

**Affiliations:** ^1^Lymphatic Filariasis Support Centre, School of Public Health, Tropical Medicine and Rehabilitation Sciences, James Cook University, Townsville, QLD 4811, Australia; ^2^World Health Organization, Apia, Samoa; ^3^Ministry of Health, Apia, Samoa; ^4^National Health Service, Apia, Samoa; ^5^Vector Borne Disease Unit, Ministry of Health, Port Vila, Vanuatu; ^6^Ministry of Health, P.O. Box 59, Nuku'alofa, Tonga; ^7^World Health Organization, 50490 Kuala Lumpur, Malaysia

## Abstract

Elimination of lymphatic filariasis (LF) in the Pacific Island Countries and Territories (PICT) has been defined as <0.1% circulating filarial antigen (CFA) prevalence in children born after the implementation of successful mass drug administrations (MDAs). This research assessed the feasibility of CFA and antibody testing in three countries; Tonga, Vanuatu, and Samoa. Transmission is interrupted in Vanuatu and Tonga as evidenced by no CFA positive children and a low antibody prevalence and titre. Transmission is ongoing in Samoa with microfilaraemic (Mf) and CFA positive children and a high antibody prevalence and titre. Furthermore, areas of transmission were identified with Mf positive adults, but no CFA positive children. These areas had a high antibody prevalence in children. In conclusion, CFA testing in children alone was not useful for identifying areas of residual endemicity in Samoa. Thus, it would be beneficial to include antibody serology in the PICT surveillance strategy.

## 1. Introduction

Lymphatic filariasis (LF), a mosquito-transmitted parasitic disease caused by the filarial nematodes *Wuchereria bancrofti*, *Brugia malayi,* and *Brugia timori*, is classified as a neglected tropical disease (NTD) that is endemic in many parts of the world including the South Pacific [1–3]. The Global Elimination Programme to eliminate LF (GPELF) began in the late 1990s, and the Pacific counterpart of GPELF, formed in 1999 under the auspices of the World Health Organization (WHO), was named the Pacific Programme for the Elimination of Lymphatic Filariasis (PacELF) [4, 5]. PacELF resolved to eliminate LF as a public health problem in the Pacific using mass drug administrations (MDAs) [5]. Elimination of LF was defined as <1% circulating filarial antigen (CFA) prevalence of the population and <0.1% CFA prevalence in children born after the implementation of MDAs [6].

Sixteen of the 22 countries falling under the jurisdiction of PacELF were classified as endemic for LF following baseline prevalence surveys. They were American Samoa, the Cook Islands, the Federated States of Micronesia, Fiji, French Polynesia, Kiribati, the Marshall Islands, New Caledonia, Niue, Palau, Papua New Guinea, Samoa, Tonga, Tuvalu, Vanuatu, and Wallis and Futuna [7]. The Cook Islands, Niue, Vanuatu, and Tonga have reached <1% CFA prevalence of the population following five rounds of MDA and are now implementing activities to ensure that transmission has been interrupted and to detect any remaining and/or new foci of transmission [8]. The challenge for this phase of the LF programme is to find a suitable strategy for surveillance in those countries that have reached the target threshold. 

Recently, child transmission surveys (CTS) have been proposed as a potential method for surveillance in the South Pacific as part of the “draft LF active surveillance strategy for the Pacific Island Countries and Territories (PICT)” [7]. This method relies on detecting CFA positive children in either school-based or community-based surveys. The strategy was based on the preapproved LF diagnostic assays available and took into consideration resource and funding constraints [7]. Upon detection of a CFA positive child, using the field immunochromatographic test (ICT), surrounding residents (200 m radius or 24 houses) would be tested to identify the microfilaraemic (Mf) positive case from the child's house of residence [7]. Since drafting the surveillance strategy, three South Pacific countries have finished their CTS, either alone or in conjunction with post-MDA prevalence surveys. The CTS was completed in two countries which had entered surveillance mode, Tonga and Vanuatu, and Samoa, where transmission was ongoing [8].

As the prevalence of LF declines, the sensitivities of the gold standards, which measure Mf and CFA, also decrease [9–13], potentially making these assays less useful during this stage of the LF elimination programme [14–16]. Antibody responses to LF exposure have been suggested as an ideal immunological measurement for identifying areas of residual endemicity in future surveillance campaigns [9, 14, 17–19]. Previous studies demonstrated that antibody production in response to LF exposure occurs during the first few years of life [20]. Therefore, children born after the cessation of LF transmission should be antibody negative [17, 21–27]. One standardised diagnostic tool for the detection of antibodies is the Filariasis Cellabs Enzyme Linked Immunosorbent Assay (CELISA). This diagnostic assay measures antifilarial IgG_4_ and is adaptable for both serum samples and filter paper samples with little interlaboratory variation [28, 29]. Antibody serology can be used in conjunction with seroprevalence mapping, which has been demonstrated to be useful for visualising problem areas where exposure occurs [30].

Filariasis control began in Tonga in 1977, and by 1999, when Tonga joined PacELF, the CFA prevalence was recorded as 2.7% (http://www.wpro.who.int/pacelf/programmes/ton/PacELF+Activities+to+Date.htm). MDA rounds were completed annually in Tonga for five years from 2001 to 2005 with reported coverage rates of 79%, 84%, 91%, 86%, and 85%, respectively, [8]. Following the fifth round of MDA, Tonga recorded an overall CFA prevalence of 0.4% and, therefore, completed its CTS in 2007. The results are presented in this paper.

By 2005, Vanuatu had completed its fifth round of MDA (http://www.wpro.who.int/pacelf/programmes/van/activities.htm). The annual rounds were from 2000 to 2004 with reported coverage rates of 83%, 84%, 84%, 87%, and 85%, respectively, [8]. Following the final round of MDA in 2005, countrywide CFA prevalence was 0.32%, with no detectable Mf positives (Vanuatu country report, *unpublished data*) [8]. Consequently, Vanuatu completed its CTS in 2007, the results of which are presented in this paper.

Samoa has a long history of filariasis control; initial filariasis surveys began as early as the 1920s, with attempts at control programmes in the 1940s [4]. In 1966, MDAs began and Samoa completed 10 rounds of MDA before the establishment of PacELF [31, 32]. In 1999, Samoa was the first country to implement the new MDA regimen using a combination of diethylcarbamazine citrate (DEC) and albendazole [4]. A further six rounds of MDA were completed from 1999 to 2006. The reported MDA coverage for the five rounds conducted from 1999 to 2003 was 90%, 57%, 68%, 60%, and 80%, respectively, [8]. In 2004, a stratified cluster nationwide survey demonstrated an overall Mf prevalence of 0.4% with a CFA prevalence of 1.1%. This corresponded to a 75.6% reduction in CFA positive individuals since the beginning of the new national programme in 1999 [4, 5]. The results from the follow-up survey in 2007 are presented in this paper in conjunction with the 2007 CTS.

The aim of this research was to determine if the presence of CFA positive children alone accurately reflected a cessation of LF transmission, as assumed in the draft surveillance strategy, or if it was necessary to complement CFA testing with antibody serology using the Filariasis CELISA. By doing so, we help contribute to defining a cessation of LF transmission and early detection of possible resurgence as part of active surveillance. This will assess the potential of the Filariasis CELISA to be incorporated into the repertoire of available LF diagnostic assays currently used in LF elimination programmes. Serological mapping could then be used to easily identify and visualise areas where exposure is ongoing and where further investigations could be required by the country LF programme managers [33]. 

## 2. Materials and Methods

### 2.1. Study Population

Three South Pacific countries, Samoa, Tonga, and Vanuatu, participated in the study in 2007. The study was conducted under Human Ethics Approval number H1423, as approved by the James Cook University Research Human Ethics Committee.

#### 2.1.1. Samoa

The post-MDA prevalence survey was a national survey conducted by health district, across both Savai'i and Upolu using the stratified cluster method. Stratified cluster sampling was performed by dividing the country into implementation units (IUs) within which villages were chosen randomly [6]. Within the village, a minimum of five households were randomly chosen. Participants of any age were asked to register, and blood samples were taken. All participants were bled for ICT testing and those that tested ICT positive were rebled for Mf testing. Filter paper samples were also collected from children aged 5 to 10 years for antibody serology. Overall, the survey included 6648 people, with 2315 children ≤10 years. Of these children, 1045 were included in antibody testing.

#### 2.1.2. Tonga

CTS carried out in Tonga was in accordance with the “draft LF active surveillance strategy for the PICT” using the school-based approach [7], but with the addition of antibody serology. Blood collection occurred in primary schools across the islands of ‘Eua, Ha'apai, and Vava'u. Overall, 797 children aged 5 to 6 years participated in the study. All participants were bled for ICT testing and those that tested ICT positive were rebled for Mf testing. Filter paper samples were also collected for antibody serology.

#### 2.1.3. Vanuatu

A total of 3840 children aged 5 to 6 years were bled across the country in accordance with the CTS outlined in the “draft LF active surveillance strategy for the PICT” using the community-based approach [7], but with the addition of antibody serology. These children were bled for ICT and those positive were bled for Mf testing. Filter paper samples were also collected for antibody serology.

### 2.2. Blood Collection

Following registration and informed verbal consent, approximately 220 *μ*L of blood was collected by fingerprick. One hundred microlitres was used for CFA testing in the field. If the ICT test was positive, 60 *μ*L was used to make a three-line thick blood smear for Mf examination. The remaining blood was soaked onto the six protrusions of a Tropbio filter paper disc, each holding 10 *μ*L of blood (Tropbio Pty Ltd, QLD, Australia). The filter paper disc was dried, placed in ziplock bags, and transported back to Australia for storage at −20°C until testing for antifilarial antibodies.

### 2.3. Circulating Filarial Antigen Testing

The field test to detect CFA was the NOW filariasis Immunochromatographic test (ICT) and performed according to the manufacturer's instructions as previously described (Binax, Portland, ME, USA) [34]. Briefly, the collected 100 *μ*L of blood was transferred onto the absorbent pad using the capillary tube and the result was read at exactly 10 minutes, according to the manufacturer's instructions, and recorded as positive, negative, or invalid. Positives (referred to as CFA positive) were rebled for Mf examination.

### 2.4. Microfilaremia Testing

Blood taken from a fingerprick was smeared into three lines, approximately 20 *μ*L thick, onto a microscope glass slide using a capillary as previously described [35]. The slides were left to dry for 48 hours then wrapped for transport. In the laboratory of each country, each slide was stained in 10% Giemsa stain (20 minutes), washed in water, dried, then coverslipped. The slide was examined under the microscope (×200), and Mf were recorded. The number of Mf per mL of blood was calculated based on the initial 60 *μ*L volume. Blood collection for Mf testing in Vanuatu and Tonga occurred between 2200 and 0100 hours. Blood collection for Mf testing in Samoa occurred between 0800 and 2000 hours according to peak levels of Mf and biting tendencies of *Aedes polynesiensis* [36].

### 2.5. Antibody Testing with the Filariasis CELISA

Antifilarial IgG_4_ antibodies were detected using the Filariasis CELISA kit (Cellabs Pty Ltd, Manly, Australia). One protrusion of filter paper was eluted overnight at 4°C in 500 *μ*L of sample diluent. The following morning, the elution was thoroughly vortexed and assayed in duplicate, according to the manufacturer's instructions. The washing steps were performed with an automated plate washer (MultiDrop Combi nL, Pathtec, VIC, Australia) using 200 *μ*L per well. Plates were read at a dual wavelength of 450 nm and 650 nm with a Multiskan EX Type 355 Primary V.2.1-0 (Pathtec, VIC, Australia) using the software Labsystems Genesis Version 3.00 (Pathtec, VIC, Australia). Negative samples were defined as OD value <0.260, and positive samples were defined as OD value ≥0.400 [28]. Samples with values between these OD values were repeated, in accordance with the manufacturer's instructions, and if the OD value was <0.400, they were considered negative. Antibody prevalence was based on positive/negative reactivity of the samples. Antibody titres were assumed to correspond to the OD absorbance value [37]. 

### 2.6. Statistical Analysis and Prevalence Mapping

All data was entered into SPSS Statistical Software Package Version 17.0. Prevalence rates were calculated using the descriptive options in SPSS, and 95% Confidence Interval (CI) was calculated using the JavaStat binomial software (http://statpages.org/confint.html) and included in the figures. Chi-squared test was used to investigate the difference in prevalence rates among countries and health districts in Samoa. The Kruskall-Wallis test for nonparametric data was used to compare the OD absorbance values (antibody titres) among the three countries. Prevalence of Mf, CFA, and antibody in Samoa was mapped using CorelDRAW. 

## 3. Results

### 3.1. Study Population

**Figure 1:**
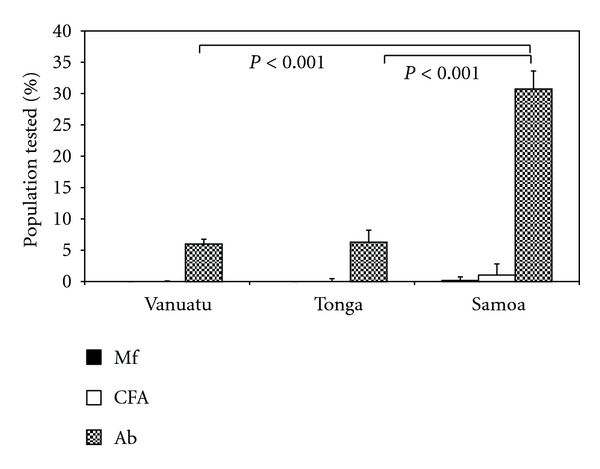
Prevalence rates of the diagnostic parameters: Mf, CFA, and antibody in children. There was a significantly lower antibody (Ab) prevalence in children in Vanuatu and Tonga and a significantly higher antibody prevalence in children in Samoa (*χ* ^2^ = 549.3; *df* = 3; *P* < 0.001). There were no Mf or CFA positive children detected in either Vanuatu or Tonga.

**Figure 2:**
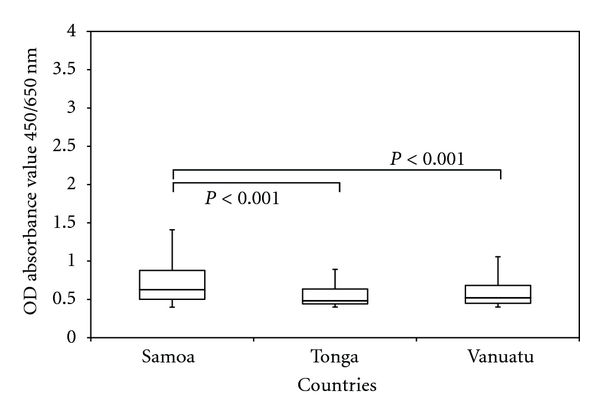
Differences in antibody titre in antibody-positive children residing in Samoa, Tonga, and Vanuatu. Of those children who were antibody positive, there was a significantly higher average antibody titre in Samoa than Vanuatu and Tonga (*χ* ^2^ = 41; *df* = 2; *P* = 0).

In Vanuatu and Tonga, all children tested negative for CFA, whereas in Samoa there were Mf and CFA positive children detected (Figure 1). This was coupled with a significantly lower antibody prevalence recorded for both Vanuatu (6.0%) and Tonga (6.3%) when compared to Samoa (30.7%) (*P* < 0.001, Figure 1). In addition, of those children who were antibody positive, there was a significantly higher average antibody titre in Samoa than Vanuatu and Tonga (*P* = 0, Figure 2). Consequently in Samoa, the overall significantly higher antibody prevalence and titre, coupled with Mf and CFA positive children, was further analysed by health district to identify potential residual areas of endemicity. 

#### 3.1.1. Samoa

**Figure 3:**
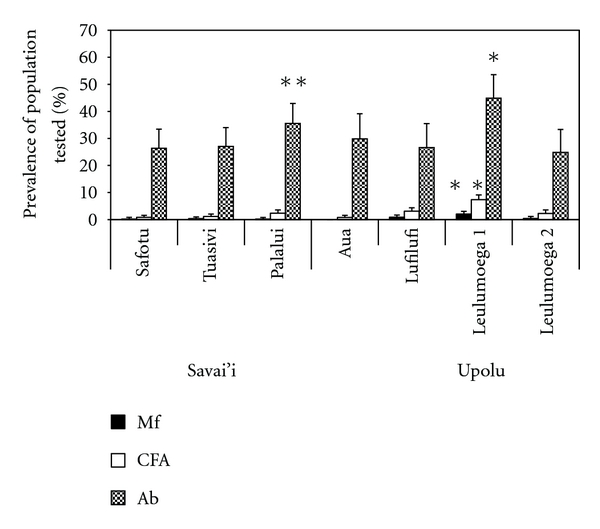
Mf, CFA, and antibody prevalence across the health districts in Samoa. Leulumoega 1 recorded a significantly higher Mf (∗), CFA (∗), and antibody (Ab) prevalence in children (∗) than the other districts (*χ* ^2^ = 46.9; *df* = 6; *P* < 0.001), (*χ* ^2^ = 124.9; *df* = 6; *P* < 0.001), (*χ* ^2^ = 20.6; *df* = 6; *P* < 0.001). Similarly, Palalui recorded a significantly high antibody (Ab) prevalence (∗∗) in children (*χ* ^2^ = 20.6; *df* = 6; *P* < 0.001).

**Figure 4:**
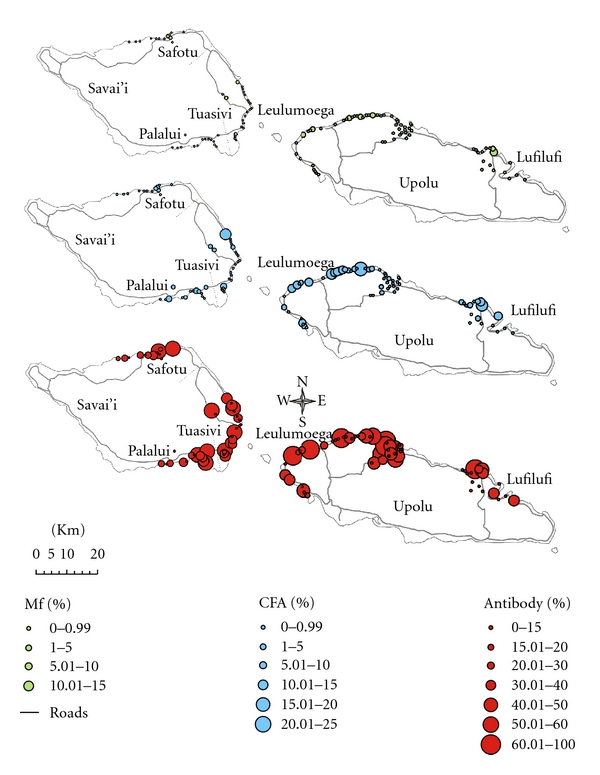
Serological mapping for Mf, CFA, and antibody prevalence across Samoa. Geographical mapping allows quick reference to suspected problem areas or “hot spots” of residual endemicity.

When analysing the data by health district, Leulumoega 1, on Upolu, had a significantly higher Mf prevalence (2%) (*P* < 0.001) and CFA prevalence (7.3%) (*P* < 0.001, Figure 3). Furthermore, including Palalui, Leulumoega 1 had a significantly higher antibody prevalence in children (44.9%) (*P* < 0.001, Figure 3). There were three health districts with detectable Mf positive adults, but no CFA positive children (Safotu (0.2%) and Tuasivi (0.3%) on Savai'i and Leulumoega 2 (0.4%) on Upolu). In these areas, Mf prevalence exceeded 0.1%, the defined threshold for ongoing transmission [6], and the antibody prevalence in children exceeded 20%. Prevalence for the three parameters was easily visualised on the geographical maps, including residual endemic areas in the aforementioned districts (Figure 4). 

**Figure 5:**
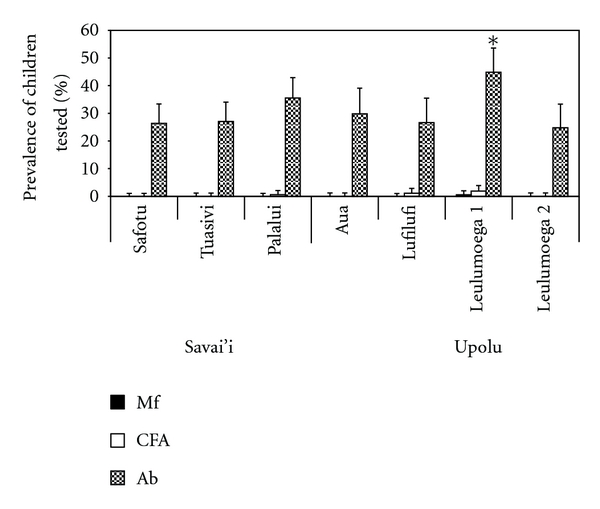
Mf, CFA, and antibody prevalence in children ≤10 years only across the health districts in Samoa. Similar to the data across all ages groups, Upolu had a significantly higher CFA prevalence in children than Savai'i (*χ* ^2^ = 4.086; *df* = 1; *P* = 0.043). No differences for Mf prevalence were observed between the two island groups (*χ* ^2^ = 1.514; *df* = 1; *P* = 0.218). Similarly, no significant differences for Mf prevalence were observed among the health districts (*χ* ^2^ = 10.694; *df* = 6; *P* = 0.098), since Mf positive children were identified only in Leulumoega 1. Complementing this result, Leulumoega 1 recorded a significantly higher antibody (Ab) prevalence (∗) than the other districts (*χ* ^2^ = 20.6; *df* = 6; *P* < 0.001). The health districts of Aua, Leulumoega 2, Safotu, and Tuasivi recorded no CFA positive children. There were no significant differences for CFA prevalence in children among the health districts (*χ* ^2^ = 2.612; *df* = 2; *P* = 0.271).

When analysing the data for children only, Upolu maintained a significantly higher CFA prevalence (*P* = 0.043), yet no differences were observed for Mf prevalence between the islands (*P* = 0.218, Figure 5). For the health districts, Mf positive children were found only in Leulumoega 1, which had a significantly higher antibody prevalence (Figure 3) and the highest CFA prevalence, although not significantly higher than the other villages with CFA positive children (*P* = 0.271). Lufilufi and Palalui were the other 2 villages with CFA-positive children (Figure 5).

## 4. Discussion and Conclusions

Active LF surveillance requires accurate and sensitive diagnostic assays to detect residual areas of endemicity and resurgence early. The current draft LF active surveillance strategy for the PICT [7] relies solely on CFA testing in children, since the use of antibody serology is not yet currently approved [6]. This is due to the need for standardisation, which can now be achieved using the commercially available Filariasis CELISA (Cellabs Pty Ltd). It was the aim of this research to assess the validity of the current surveillance strategy and if incorporating antibody serology would be advantageous in the PICT. The first aim of the research was partially addressed, but until long-term monitoring of the outcomes of the elimination programme occurs, the strategy will continue to be dynamic to meet individual country needs.

The data indicated the cessation of LF transmission in both Tonga and Vanuatu and ongoing LF transmission in Samoa. In Tonga and Vanuatu, there were no detectable CFA positive children coupled with a significantly low antibody prevalence and titre. Identification of Mf and CFA positive children in Samoa was coupled with a significantly higher antibody prevalence and titre. For Vanuatu and Tonga, the higher value of antibody prevalence (6%) versus the CFA prevalence (0%) is a reflection of the sensitivity and specificity of the Filariasis CELISA for filter paper sampling. When using filter paper samples, the Filariasis CELISA has a positive predictive value (PPV) of 60% (95% CI = 43–75) [28]. This means that a false positive is expected at a rate of approximately 40% (95% CI = 25% to 57% of the time) [28]. Filter paper samples have inherent higher OD values [38], but it would be disadvantageous to use serum for wide-scale programmatic sampling because of the difficulties with collection and storage [28]. The higher PPV of the Filariasis CELISA does not impact on the usefulness of the diagnostic assay from a programmatic perspective, since recent research has demonstrated the use of the assay for identifying clusters of exposed children [39]. It could be suggested that it would be appropriate for the country programme managers to follow up any identified clusters of exposed children with a more in-depth serological study using the gold standard serum. This would be on a smaller and more manageable scale than the programmatic survey.

Previous studies in Egypt, where the vector *Culex sp*. is endemic [40], suggest a <2% antibody prevalence in first-year primary school children as a threshold for elimination [25]. This study did indicate the potential necessity for different threshold targets for other endemic areas. The data presented here does not concur with a 2% antibody prevalence threshold for interruption of transmission in the PICT since both Vanuatu and Tonga recorded prevalence rates of 6% and transmission was interrupted. However, the studies in Egypt used serum samples with laboratory-based ELISAs, not filter paper samples with the commercial Filariasis CELISA.

The detection of ongoing transmission in Samoa was further analysed by each health district to assess if the current surveillance strategy could identify problem areas. CFA testing alone in children (CTS) was not adequate for identifying all of the key residual areas of endemicity in Samoa. There were three health districts with detectable Mf positive adults, but no CFA positive children, where transmission would likely be occurring. This is because in these health districts Mf prevalence exceeded 0.1%, the recommended threshold by WHO for the interruption of transmission [6] and spatial clustering of LF has been previously identified [39]. The health districts identified were Safotu and Tuasivi on the island of Savai'i and Leulumoega 2 on the island of Upolu. In these health districts, the antibody prevalence in children exceeded 20%, an observation compatible with ongoing exposure. Very importantly, it must be emphasised that these health districts would not have been detected as areas of residual or reemerging infection based on CFA testing in children alone and would have been missed if the proposed WHO surveillance programme using ICT testing was in place. These significant foci were only identified because antibody serology was included in the testing protocol. Therefore, it would be beneficial to strengthen the current strategy surveillance strategy by including antibody serology.

In Samoa, the identification of Mf and CFA positive children is indicative of ongoing transmission, which is further evidenced by a significantly higher antibody prevalence and titre in children. These findings support previous studies whereby Samoa exceeded 1% CFA prevalence following five rounds of MDA [8]. The reasons for ongoing transmission in Samoa are yet to be ascertained, but it is crucial that these are addressed in order for LF elimination to be successful. It has been suggested that lack of directly observed therapy (DOT), lack of compliance, and lack of vector control could all be contributing factors [8, 31, 41, 42]. Recent research has indicated the potential for vector control in Samoa which will have a positive impact on the Samoan LF elimination programme [39]. The seroprevalence maps in the current study could provide the Samoan LF programme managers with a starting point to further investigate residual endemic areas and potentially incorporate vector control. 

In addition, the results from the current study indicate that antibody prevalence thresholds need to be complemented with analysing antibody titres, since the low antibody prevalence in Tonga and Vanuatu was coupled with a significantly low antibody titre in positive children. Collectively, low prevalence and low titre (with no detectable antigen-positive children) suggest an interruption of transmission. During active surveillance mode, the detection of an area with low antibody prevalence but high antibody titre could require further investigation.

In conclusion, transmission of LF has been interrupted in Tonga and Vanuatu as evidenced by no detectable CFA positivity in children and significantly low antibody prevalence and titres. Ongoing transmission of LF is evident in Samoa based on the presence of Mf/CFA positive children and a significantly high antibody prevalence and titres. Most importantly, key residual areas of endemicity, which can also be observed by seroprevalence mapping, would have been overlooked when testing for CFA positivity alone in children. This holds promise for future incorporation of antibody prevalence mapping in the current surveillance strategy for the PICT.
